# Assessment of lung function and severity grading in interstitial lung diseases (% predicted versus *z*-scores) and association with survival: A retrospective cohort study of 6,808 patients

**DOI:** 10.1371/journal.pmed.1004619

**Published:** 2025-05-29

**Authors:** Piotr W. Boros, Magdalena M. Martusewicz-Boros, Katarzyna B. Lewandowska

**Affiliations:** 1 Lung Pathophysiology Department, National TB and Lung Diseases Research Institute, Warsaw, Poland; 2 3rd Lung Diseases and Oncology Department, National TB and Lung Diseases Research Institute, Warsaw, Poland; 3 1st Lung Diseases Department, National TB and Lung Diseases Research Institute, Warsaw, Poland; Harvard Medical School, UNITED STATES OF AMERICA

## Abstract

**Background:**

Pulmonary function tests (PFTs) are essential for predicting outcomes in interstitial lung disease (ILD). In 2022, an expert panel recommended using *z*-scores instead of the traditional % predicted cut-off values to interpret the severity of PFT abnormalities which may lead to discordant classifications in some patients. To assess the magnitude and prognostic impact of this phenomenon we compared these two approaches in predicting all-cause mortality in a large cohort of patients with ILDs.

**Methods and findings:**

We retrospectively analyzed data from a tertiary referral center for patients with ILDs. Absolute FEV_1_, FVC, TLC, and TLCO values from patients’ first presentations were transformed and presented as % predicted and *z*-scores using the most recent global lung initiative (GLI) reference values. Results were categorized for severity according to % predicted and *z*-score levels. Predictors of all-cause mortality over a 14-year follow-up were determined using Kaplan–Meier survival analysis and Cox proportional hazards regression. Between January 2009 and March 2023, 6,808 patients with ILDs were evaluated at the National TB and Lung Diseases Research Institute in Warsaw, Poland. Most were diagnosed with sarcoidosis, fibrotic ILD, or non-fibrotic ILD. At their first presentation, 13.2% had airway obstruction, 23.1% had low FVC (indicative of restriction by spirometry), and 45.6% had a reduced lung transfer factor (TLCO). Reclassification of spirometric indices occurred in 26.8% of patients for FEV_1_ and 24.6% for FVC among those with abnormal results, with most being reassigned to a less severe categories. For TLCO, 28.1% of patients with reduced values were reclassified, with most shifting to more severe categories. During the follow-up, 1,525 (22.4%) of patients died. Both low FVC and low TLCO predicted all-cause mortality, with *z*-score thresholds showing stronger associations with mortality. A one-unit decrease in the FVC *z*-score was associated with a 10.3% increase in the risk of death, while a one-unit decrease in TLCO *z*-score was linked to an over 30% increase in mortality risk. Limitations of this retrospective single-center study include lack of data on cause-specific mortality, potential residual confounding, and limited generalizability to non-Caucasian or younger populations.

**Conclusions:**

The recently recommended use of *z*-scores leads to significant reclassification of lung function results in patients with ILDs, largely driven by age. This approach is justified by its stronger prognostic associations. Severe TLCO impairment remains a robust predictor of mortality in ILDs.

## Author summary

**Why was this study done?:**

- Lung function measurements are essential for predicting outcomes in interstitial lung diseases, and impairment severity is traditionally categorized using % predicted values.
- The adoption of *z*-scores, as recommended by ATS/ERS experts, significantly changes the classification of impairment severity for a substantial number of patients.
- This study aimed to determine which classification system better predicts mortality risk in patients with interstitial lung diseases.

**What did the researchers do and find?:**

- We retrospectively analyzed data from 6,808 patients with interstitial lung diseases over a 14-year period and compared impairment classifications at first presentation using traditional % predicted values and the new *z*-score-based system.
- The *z*-score-based system reclassified lung function severity in 25% of patients for ventilatory impairment (to a less severe category) and in 28% for lung transfer factor impairment (to a more severe category), with age being the primary factor influencing reclassification.
- The *z*-score-based classification demonstrated better accuracy in predicting mortality risk compared to the % predicted-based system in Cox proportional hazards regression models.

**What do these findings mean?:**

- Implementing the *z*-score-based system reclassifies older patients to less severe ventilatory impairment categories and younger patients to more severe lung transfer factor impairment categories.
- Lung transfer factor impairment remains a strong predictor of mortality in interstitial lung diseases.
- These findings support the potential of a *z*-score-based classification system for prognostic purposes; however, as this is a retrospective study, prospective studies and clinical trials are needed to validate these results.

## Abstract 3

Piotr W. Boros and colleagues determine which classification system, % predicted values or z-score based, better predicts mortality risk in patients with interstitial lung disease.

## Introduction

Lung function measurements are essential for predicting outcomes of interstitial lung diseases (ILDs). Results from spirometry measurements (e.g., forced vital capacity, FVC) and gas transfer tests (e.g., lung transfer factor for carbon monoxide, TLCO) are used as components of well-known and frequently used scales, such as the ‘GAP Index’ or ‘du Bois Score’ in idiopathic pulmonary fibrosis [1,2]. However, it is the TLC (total lung capacity) value that determines the presence of restrictive disorders, and FEV_1_ (forced expiratory volume in one second) is considered a universal indicator of the severity of disorders, regardless of whether they are of obstructive or restrictive origin. For many years following the ATS (American Thoracic Society) and ERS (European Respiratory Society) 2005 recommendations, a *z*-score threshold of −1.64 (level of the 5th percentile) was an established criterion for classifying results as abnormal, and this approach was well accepted within the respiratory community [3]. It functioned in parallel with the classification of lung function impairment severity based on % predicted values, where different % predicted thresholds were applied to ventilatory disorders (e.g., 50% for severe/non-severe) and different ones to TLCO (40% pred for severe/non-severe).

The recently published recommendations for routine lung function interpretative strategy suggest abandoning % predicted and use of *z*-scores as more appropriate also for severity assessment [4]. One of the arguments is to better match the risk of death [5,6]. However, the ATS/ERS experts in the rationale refer either to chronic obstructive pulmonary disease (COPD) [5], or to a cohort of several thousand patients, in which a clearly defined diagnosis was available for only 38% of cases, with interstitial lung diseases (ILD) accounting for just 9% [6].

In contrast to the referenced study, where more than half of the patients had no confirmed diagnosis and received no treatment, a key strength of our study is that in the analyzed large cohort (*n* = 6,808), all patients had a confirmed diagnosis belonging to the group of interstitial lung diseases. This enabled us to test our hypothesis in a well-defined population of patients with established diagnoses, representing a group of relatively rare diseases, but associated with a serious prognosis (excluding sarcoidosis), where the role of pulmonary function parameters has always been considered. Previously published data indicated, that changes in the cut-off points may lead to discordance in classification in some patients [7]. From a practical and clinical point of view, it seems important to answer the question of whether the change in the method of evaluating test results and thus reclassifying the severity in some patients will actually result in a better match to their prognosis.

The main objective of the analysis was to examine the relationship between the presence and severity of various lung function disorders (ventilation and gas exchange) with consideration of two different assessment methods (ATS/ERS 2005 and ATS/ERS 2022) in the context of prognosis in a large group of patients with ILD. We hypothesized that there were significant differences in prognosis in patients with inconsistent classification compared to their original severity grade.

## Materials and methods

### Ethics statement

This study is a retrospective analysis of de-identified data. All procedures performed in the study were in accordance with the ethical standards of the institutional and national research committee and with the 1964 Helsinki declaration and its later amendments. The study was reviewed and approved by our institutional bioethics committee at the National TB and Lung Diseases Research Institute (approval number KB-80/2021), and the requirement for obtaining informed consent was waived.

### Data source and study population

We conducted a retrospective analysis of pulmonary function tests (PFTs) in a cohort of patients diagnosed with the most prevalent ILDs: sarcoidosis, hypersensitivity pneumonitis, fibrotic ILDs (e.g., idiopathic pulmonary fibrosis) and other diseases classified as ILDs, e.g., connective tissue disorders (CTD) with lung involvement, pneumoconiosis, drug-induced interstitial lung disorders. The study was conducted at a reference center for lung diseases. The clinical data comprised consecutively collected test results from patients referred for lung function assessment for the typically wide range of clinical purposes in ILDs seen in large hospital-based clinical laboratories. Tests were conducted between January 2009 and March 2023. All spirometry, lung volume, and gas transfer measurements were performed in accordance with international standards applied at the time of data collection [8–12]. This study did not have a prospectively written protocol or analysis plan. The analysis was planned in its current form at the end of 2021, following the publication of the updated ATS/ERS recommendations on the interpretation and classification of pulmonary function impairment severity. Clinical data verification and acquisition of mortality information from a governmental registry continued until March 2023. The peer-review process did not result in any substantial changes to the study’s scope or methodology.

### Lung function and outcome assessment

Only baseline (at first presentation) and pre-bronchodilator data was included. TLCO results were corrected for hemoglobin. Absolute FEV_1_, FVC, TLC and TLCO values from these patients were transformed, and we used reference values from the GLI project for all measurements (calculation made in May 2023, after correction for TLCO) [13–16], defining the lower limit of normal (LLN) at predicted −1.645 SD, and grading severity according to the ATS 1991, ATS/ERS 2005 and 2022 PFT interpretation guidelines [3,4,17].

For clarity and simplification, the ATS/ERS 2005 category of “moderately severe” was merged into the “moderate” category (ranging from 50% to 70% of predicted for FEV_1_ and FVC), and the category of “very severe” was merged into the “severe” category (<50% predicted for FEV_1_ and FVC). The category of “moderately severe” for TLC (<60% of pred.) was assigned to the “severe” category – see Table 1. Mortality data were obtained from the Ministry of Digital Affairs. Date of censoring was the March 15th 2023 and time to event was time from the first accessible pulmonary function test to censoring or death.

**Table 1:** Categorization of lung function impairment severity recommended by ATS/ERS 2022 [4] and previously in 2005 [3] and ATS 1991 [17] guidelines.

Severity stages	ATS/ERS 2022 for all indices	ATS/ERS 2005 for FEV_1_ (also applied for FVC)	ATS/ERS 2005 for TLCO	ATS 1991 for TLC
**Normal**	*z*-score > −1.645	>−1.645	>−1.645	>−1.645
**Mild**	−2.5 ≤ *z*-score ≤ −1.645	>70% pred.	>60% pred.	>70% pred.
**Moderate**	−4 ≤ *z*-score < −2.5	60%–70% pred. (moderate) 50%–60% pred. (moderately severe)	40%–60% pred.	60%–70% pred.
**Severe**	*z*-score < −4	35%–50% pred. (severe) <35% pred. (very severe)	<40% pred.	<60% pred. (moderately severe)

% pred. – % of predicted value; ATS – American Thoracic Society; ERS – European Respiratory Society; FEV_1_ – forced expiratory volume in 1 second; FVC – forced vital capacity; TLC – total lung capacity from body plethysmography; TLCO – lung transfer factor for carbon monoxide; *z*-score – the number of standard deviations from the mean value of the healthy GLI reference population.

In ILDs, particularly in progressive fibrotic diseases with a significantly poorer prognosis, unlike in obstructive diseases, greater emphasis is placed on the FVC and TLCO indices [18], rather than on FEV_1_, as is the case with asthma or COPD. Therefore, this analysis primarily focuses on these two indices. However, an examination of TLC and FEV_1_, as recommended for the assessment of restrictive disorders and the extent of ventilatory disorders in general, was also conducted.

### Statistical analyses

The Pearson Chi-squared test was used to check for differences in the prevalence of observations. Survival was estimated by the Kaplan–Meier method. The Cox proportional-hazards model was used for investigating the association between the survival time of patients and predictor variables: age, BMI, sex, disease type and lung function severity. The aim of the Cox model was predictive inference rather than causal inference. The variables included are those commonly considered significant risk factors for mortality in patients with ILD and have therefore been previously used as components of mortality risk scores (such as the ‘GAP Index’ [1] or the ‘du Bois Score’ [2]). The hazard ratios with 95% confidence intervals and *P*-values are presented. All statistical analyses were performed using MedCalc Statistical Software version 20.218 (MedCalc Software Ltd, Ostend, Belgium; https://www.medcalc.org; 2023). This study is reported as per the Strengthening the Reporting of Observational Studies in Epidemiology (STROBE) guideline (S1 File STROBE Checklist).

## Results

### Baseline characteristics of the patients

During the 14-year study period, over twenty-two thousand patients were investigated in our PFT lab. From these, we identified 6,808 patients (in whom 3281–48.2% were females) with diagnosed ILD. All of them were Caucasians. For the purposes of this study, we grouped patients according to the latest available information on their diagnoses into the following categories: sarcoidosis (SAR), idiopathic pulmonary fibrosis (IPF), unclassifiable interstitial lung disease (u-ILD), idiopathic non-specific interstitial pneumonia (i-NSIP), hypersensitivity pneumonitis (HP), connective tissue diseases pulmonary related disorders (CTD) and other ILDs (o-ILDs). See Table 2 for detailed patient characteristics with the breakdown of the data for each diagnosis group.

**Table 2:** Study group characteristics with the breakdown of the data for each diagnosis group. All were Caucasian. Mean ± standard deviation is given for continuous variables. Airway obstruction: FEV_1_/FVC < LLN, volume restriction: TLC < LLN, mixed: FEV_1_/FVC < LLN and TLC < LLN, non-specific: FEV_1_/FVC > LLN, TLC > LLN, FEV_1_ < LLN, FVC < LLN.

	Diagnosis group							
	SAR	CTD	HP	i-NSIP	IPF	o-ILD	u-ILD	All
*n=*	3,180	640	515	128	757	1,391	197	6,808
Age (years)	43.26 ± 11.77	59.56 ± 12.5	52.34 ± 12.96	57.51 ± 13.61	68.3 ± 9.64	53.44 ± 15.73	61.37 ± 12.2	51.13 ± 15.3
BMI (kg/m^2^)	27.54 ± 4.88	27.33 ± 4.97	28.01 ± 5.07	28.36 ± 4.15	28.57 ± 4.27	27.06 ± 5.33	28.2 ± 4.93	27.61 ± 4.94
FEV_1_ (% pred.)	90.7 ± 17.33	82.22 ± 18.31	76.58 ± 19.63	80.57 ± 20.92	87.47 ± 18.19	84.87 ± 21.23	85.17 ± 20.62	86.94 ± 19.2
FEV_1_ (*z*-score)	−0.68 ± 1.29	−1.21 ± 1.26	−1.68 ± 1.42	−1.36 ± 1.49	−0.79 ± 1.16	−1.08 ± 1.52	−1 ± 1.42	−0.92 ± 1.37
FEV_1_/FVC (absolute)	0.77 ± 0.09	0.79 ± 0.09	0.8 ± 0.09	0.82 ± 0.07	0.8 ± 0.08	0.76 ± 0.11	0.8 ± 0.08	0.78 ± 0.09
FEV_1_/FVC (*z*-score)	−0.57 ± 1.16	0 ± 1.16	0.14 ± 1.23	0.42 ± 0.95	0.4 ± 1.04	−0.44 ± 1.45	0.3 ± 1.12	−0.29 ± 1.27
FVC (% pred.)	95.07 ± 14.61	82.75 ± 18.4	76.44 ± 20.17	78.34 ± 21.61	84.74 ± 18.3	88.54 ± 18.43	83.2 ± 19.92	89.36 ± 18.08
FVC (*z*-score)	−0.38 ± 1.13	−1.22 ± 1.33	−1.77 ± 1.56	−1.59 ± 1.63	−1.01 ± 1.22	−0.85 ± 1.36	−1.18 ± 1.45	−0.78 ± 1.34
TLC (% pred.)	99.76 ± 12.78	90.58 ± 18.53	86.08 ± 20.21	81.19 ± 18.7	84.41 ± 16.71	98.47 ± 17.65	87.28 ± 18.32	95.18 ± 17
TLC (*z*-score)	−0.04 ± 1.07	−0.84 ± 1.56	−1.25 ± 1.78	−1.63 ± 1.62	−1.27 ± 1.36	−0.16 ± 1.44	−1.08 ± 1.53	−0.43 ± 1.42
TLCO (% pred.)	90.52 ± 16.99	65.4 ± 21.38	60.3 ± 21.82	57.13 ± 21.04	54.92 ± 19.2	74.24 ± 22.16	64.61 ± 24.27	77.21 ± 23.81
TLCO (*z*-score)	−0.74 ± 1.28	−2.74 ± 1.94	−3.3 ± 2.1	−3.48 ± 2.07	−3.36 ± 1.75	−2.02 ± 1.87	−2.79 ± 2.16	−1.79 ± 1.96
Airway obstruction	16.4%	8.6%	8.2%	1.6%	3.6%	17.3%	5.6%	13.2%
Volume restriction	7.0%	29.5%	40.6%	49.2%	39.2%	14.9%	36.0%	18.5%
Mixed	1.6%	0.9%	0.8%	1.6%	0.0%	1.0%	0.5%	1.1%
Non-specific	2.6%	5.6%	6.6%	3.9%	2.5%	5.4%	3.0%	3.8%
Low FEV_1_	21.0%	33.6%	49.1%	45.3%	24.0%	31.2%	31.0%	27.5%
Low FVC	12.2%	35.5%	51.7%	49.2%	28.4%	24.7%	34.5%	23.1%
Low TLCO	20.8%	68.6%	78.1%	79.7%	83.5%	52.8%	66.5%	45.6%
Any PF abnormality	32.3%	94.9%	97%	99.2%	96.3%	82.1%	98.1%	54.6%

% pred. – % of predicted value; BMI – body mass index; CTD – connective tissue diseases pulmonary related disorders; FEV_1_ – forced expiratory volume in 1 second; FVC – forced vital capacity; HP – hypersensitivity pneumonitis; i-NSIP – idiopathic non-specific interstitial pneumonia; IPF – idiopathic pulmonary fibrosis; LLN – lower limit of normal; o-ILD – other ILDs; PF – pulmonary function, SAR – sarcoidosis; SD – standard deviation; TLC – total lung capacity from body plethysmography; TLCO – lung transfer factor for carbon monoxide; u-ILD – unclassifiable interstitial lung disease; *z*-score – the number of standard deviations from the mean value of the healthy GLI reference population.

Any pulmonary function disorders were identified in 54.6% of patients (see S1 Table for details for overlapping ventilatory and gas transfer abnormalities). In most patients, abnormalities in any lung function test results were observed in 82% to 99%, depending on the group, except for patients with sarcoidosis, where the percentage of any abnormalities did not exceed 33%.

### Severity classification % pred. versus *z*-scores

Adopting the 2022 PFT interpretation guidelines (using *z*-score severity thresholds instead of % predicted) resulted in reclassifying the severity of 26.8% of patients with a low FEV_1_, 24.6% of patients with a low FVC, 15.1% of patients with low TLC and 28.1% of those with a low TLCO.

**Fig 1:**
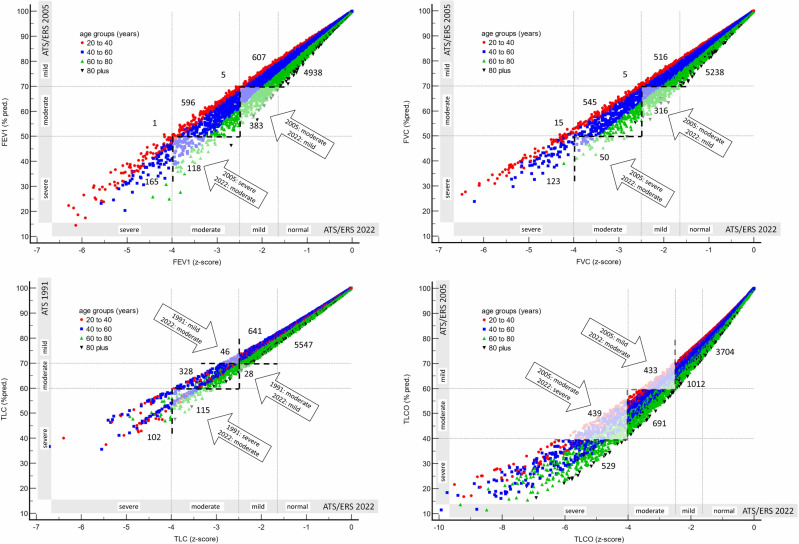
Plots of FEV_1_ (upper left), FVC (upper right), TLC (lower left) and TLCO (lower right) values expressed in % pred. (*y*-axis) against *z*-scores (*x*-axis) with subjects stratified by age group. Arrows indicate areas of discordance between the 2005, 1991 and 2022 severity categories. Horizontal dotted lines are at 2005 or 1991 severity thresholds. Dashed vertical lines are at 2022 severity thresholds. Note that results with a *z*-score above −1.645 are considered normal. The number of patients in each category is provided. For example, 5,238 patients had a normal FVC. % pred. – % of predicted value; ATS – American Thoracic Society; ERS – European Respiratory Society; FEV_1_ – forced expiratory volume in 1 second; FVC – forced vital capacity; TLC – total lung capacity from body plethysmography; TLCO – lung transfer factor for carbon monoxide; *z*-score – the number of standard deviations from the mean value of the healthy GLI reference population.

Recategorization of spirometric indices (FEV_1_ and FVC) led to a shift to a less severe category for most recategorized patients. Only 6 patients with low FEV_1_ and 20 patients with low FVC were moved to a more severe category. Using *z*-score thresholds, 384 out of 980 patients with moderately low FEV_1_ were reclassified as mild, and 118 out of 283 patients with severe FEV_1_ were assigned as moderate. Similarly, 331 out of 876 patients with moderately low FVC were reclassified as mild, and 50 out of 173 patients with severe FVC were assigned as moderate (see Fig 1, upper panels).

In terms of TLC recategorization, 28 out of 356 cases were shifted from moderate to mild, and 115 out of 217 cases were shifted from severe to moderate. However, 46 out of 688 cases were reclassified from mild to moderate. Among the 489 patients currently classified as moderate, 15 were previously classified as mild, and 115 as severe.

On the other hand, the use of *z*-score thresholds for TLCO often resulted in patients with low TLCO being shifted to a more severe category. Specifically, 433 out of 1,445 patients with a mildly low TLCO were reclassified as moderate, and 439 out of 1,130 patients previously classified as moderate were now regarded as severe (see Fig 1, lower right panel).

Older patients were more likely to be reclassified as less severe in spirometry (low FEV_1_ and FVC) when the 2022 interpretation guidelines were applied. Conversely, there was a higher occurrence of recategorization of TLCO results in women, and this was associated with a younger age group. Male patients demonstrated a lower likelihood of experiencing changes in interpretation severity compared to female patients, except for TLC recategorization, where younger females were more frequently reclassified to a more severe category, while older males were more commonly recategorized to a less severe category. Groups with a small number of reclassified cases (≤20) were excluded from the analysis. Specific changes in interpretation were found to be more prevalent based on the diagnostic category, as presented in Table 3.

**Table 3:** Characteristics of patients with abnormal lung function whose severity classification changed when the *z*-score guidelines from 2022 were applied in comparison to those who were not reclassified and stayed in 2005 category. Data for groups *n* > 30 are presented. The statistically significant differences are shaded.

	FEV_1_					
	Moderate → mild	Moderate	*P*-value	Severe → moderate	Severe	*p*-value
	*n* = 383	*n* = 596		*n* = 118	*n* = 165	
**Men (%)**	49.9	49.8	0.99	46.6	60.0	0.03
**Age**	62.8 ± 10.1	47.2 ± 12.9	<0.001	55.8 ± 9.7	39.1 ± 9.8	<0.001
**Diagnosis (% of SAR/CTD/HP/i-NSIP/IPF/o-ILD/u-ILD)**	23/15/10/3/22/20/6	33/11/17/4/7/25/3	<0.001	34/13/21/2/6/21/3	27/8/17/4/4/38/3	0.07
	**FVC**					
	**Moderate **→** mild**	**Moderate**		**Severe **→** moderate**	**Severe**	
	*n* = 316	*n* = 545		*n* = 50	*n* = 123	
**Men (%)**	44.6	50.8	0.08	42.0	56.9	0.08
**Age**	64.6 ± 9.4	49.8 ± 13.0	<0.001	62.7 ± 8.1	40.0 ± 10.0	<0.001
**Diagnosis (% of SAR/CTD/HP/i-NSIP/IPF/o-ILD/u-ILD)**	14/19/12/5/24/21/6	19/15/20/5/13/23/6	<0.001	14/12/24/6/28/12/4	8/16/33/7/7/23/6	0.01
	**TLC**					
	**Mild **→** moderate**	**Mild**		**Severe **→** moderate**	**Severe**	
	*n* = 46	*n* = 641		*n* = 115	*n* = 102	
**Men (%)**	21.7	58.5	<0.001	79.1	50	<0.001
**Age**	45.6 ± 9.6	55.9 ± 15.1	<0.001	59.2 ± 13.1	46.4 ± 12.9	<0.001
**Diagnosis (% of SAR/CTD/HP/i-NSIP/IPF/o-ILD/u-ILD)**	30/17/33/7/2/9/2	25/14/11/4/22/19/5	<0.001	5/18/16/4/32/13/11	4/19/37/10/15/11/5	0.001
	**TLCO**					
	**Mild **→** moderate**	**Mild**		**Moderate **→** severe**	**Moderate**	
	*n* = 433	*n* = 1,012		*n* = 439	*n* = 691	
**Men (%)**	42.5	55.2	<0.001	40.5	60.5	<0.001
**Age**	50.6 ± 14.4	53.6 ± 16.1	0.001	54.3 ± 14.9	61.6 ± 13.6	<0.001
**Diagnosis (% of SAR/CTD/HP/i-NSIP/IPF/o-ILD/u-ILD)**	31/13/15/2/9/27/3	39/12/7/2/13/24/4	<0.001	9/18/20/3/20/26/4	11/14/13/5/29/25/5	0.002

CTD – connective tissue diseases pulmonary related disorders; FEV_1_ – forced expiratory volume in 1 second; FVC – forced vital capacity; HP – hypersensitivity pneumonitis; i-NSIP – idiopathic non-specific interstitial pneumonia; IPF – idiopathic pulmonary fibrosis; o-ILD – other ILDs; SAR – sarcoidosis; TLC – total lung capacity from body plethysmography; TLCO – lung transfer factor for carbon monoxide; u-ILD – unclassifiable interstitial lung disease

### Survival analysis

According to data from the Ministry of Digital Affairs, the national register of population, births and deaths in Poland, 1,525 (22.4%) of our investigated patients died during the follow-up period. Patients with sarcoidosis had the best prognosis, and patients with IPF had the highest mortality rate (S1 Fig and S2–S4 Tables provide a detailed breakdown of the data).

**Fig 2:**
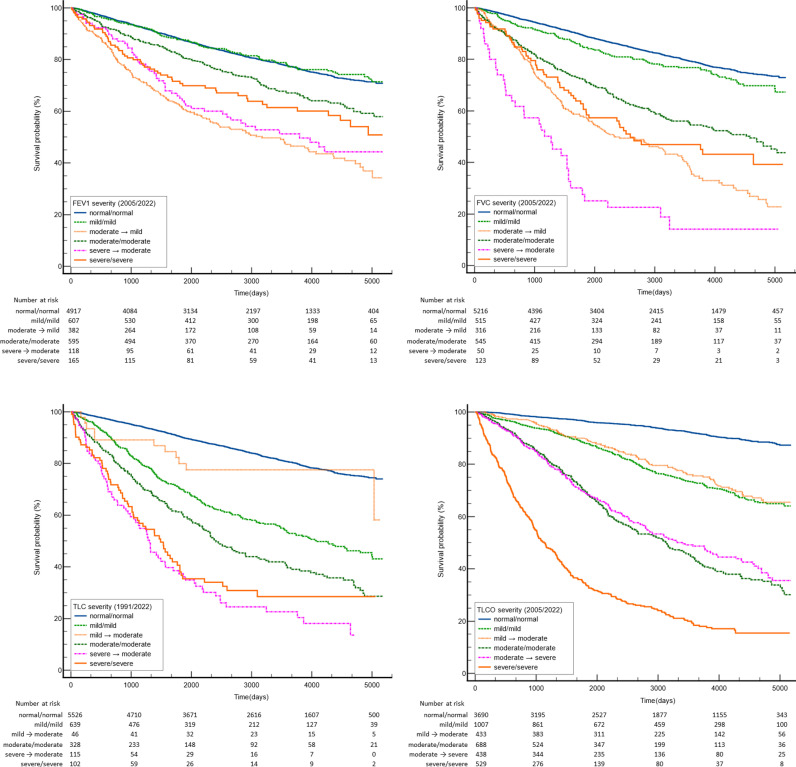
Kaplan–Meier analyses for survival according to 2005 and 1991 vs. 2022 guidelines for FEV_1_ (upper left), FVC (upper right), TLC (lower left) and TLCO (lower right) severity. Fourteen years of follow-up = 5,200 days. FEV_1_ – forced expiratory volume in 1 second; FVC – forced vital capacity; TLC – total lung capacity from body plethysmography; TLCO – lung transfer factor for carbon monoxide. Data for groups *n* > 30 are presented.

To compare the impact on prognosis between different severity levels of pulmonary function disorders, a univariate (Kaplan–Meier) model was used to examine groups of patients with concordant and discordant severity classification for predicting all-cause mortality. The results are presented in Fig 2.

In relation to ventilatory indices (FEV_1_, FVC, and TLC), an interesting observation draws attention: reclassified patients exhibited a poorer prognosis compared to those whose category remained unchanged, despite the reclassification being in the direction of milder severity. For instance, patients with a reclassified FVC from severe to moderate and from moderate to mild demonstrated a worse prognosis than those with consistently severe impairment according to both classification systems. There was a considerable overlap in prognosis among patients with moderate and severe ventilatory disorders. Regarding TLCO, the greatest separation in prognosis was observed within the groups. It was also observed that patients with a reclassified TLCO prognosis were similar to those whose baseline category did not change. The hazard ratios with 95% confidence intervals, using the group with indices in normal range as a reference, for concordant and discordant severity classes can be found in Table 4.

**Table 4:** Kaplan–Meier estimation in groups of patients recategorized according to 2022 ATS/ERS guidelines with comparison to those with concordant classification for FEV_1_, FVC, TLC and TLCO (first part of name is grade according to 2005 or 1991 classification based on % pred., the second is from ATS/ERS 2022 based on *z*-scores, results for recategorized patients are shaded). Cox proportional hazards regression was used to calculate HRs, to quantify the effect of covariates on the hazard rate.

FEV_1_ severity (2005/2022)	*n*	HR	95% CI	FVC severity (2005/2022)	*n*	HR	95% CI
Normal/normal	4,933	Reference		Normal/normal	5,233	Reference	
Mild/mild	607	0.97	0.82–1.15	Mild/mild	516	1.26	1.04–1.52
Moderate **→** mild	383	3.26	2.54–4.17	Moderate → mild	316	4.4	3.34–5.80
Moderate/moderate	596	1.57	1.31–1.87	Moderate/moderate	545	2.7	2.22–3.28
Severe **→** moderate	118	2.71	1.82–4.06	Severe → moderate	50	8.77	3.82–20.12
Severe/severe	165	2.12	1.49–3.02	Severe/severe	123	3.55	2.31–5.45
**TLC severity (1991/2022)**				**TLCO severity (2005/2022)**			
Normal/normal	5,542	Reference		Normal/normal	3,703	Reference	
Mild/mild	641	2.99	2.49–3.60	Mild/mild	1,010	3.57	3.09–4.12
Mild → moderate	46	1.38	0.77–2.48	Mild → moderate	433	3.3	2.71–4.03
Moderate → mild	28	12.19	3.59–41.34	Moderate/moderate	690	8.98	7.46–10.79
Moderate/moderate	328	4.46	3.43–5.79	Moderate → severe	439	8.33	6.68–10.39
Severe → moderate	115	8.18	4.75–14.10	Severe/severe	528	21.32	16.58–27.42
Severe/severe	102	6.82	3.96–11.72				

ATS – American Thoracic Society; CI – confidence interval; ERS – European Respiratory Society; FEV_1_ – forced expiratory volume in 1 second; FVC – forced vital capacity; HR – hazard ratio; TLC – total lung capacity from body plethysmography; TLCO – lung transfer factor for carbon monoxide;

To examine how specified risk factors affect all-cause mortality, we used the Cox proportional hazards models which included sex, age, body mass index (BMI), the diagnosis group (sarcoidosis as the reference) and lung function. Table 5 presents models with a threshold approach to the severity of the lung function disorders for both classification methods for FVC and TLCO.

**Table 5:** Hazard ratios (HR) for all-cause mortality with 95% confidence intervals from Cox regression models using the 2005 vs. the 2022 guidelines for grading severity, normal FVC (*n* = 5,238) and normal TLCO (*n* = 3,704) as reference for lung function severity, sarcoidosis as reference for other diagnosis groups. Variables which were statistically significant predictors for that model are shaded.

Covariate/factor		HR (95% CI)	*p*-value		HR (95% CI)	*p*-value
	Model with FVC severity using 2005 guidelines (% predicted thresholds)			Model with FVC severity using 2022 guidelines (*z*-score thresholds)		
**FVC “mild”**	(>70% pred., *n* = 521)	1.12 (0.91–1.37)	0.2865	(*z*–score > –2.5, *n* = 832)	1.17 (1.02–1.35)	0.0259
**FVC “moderate”**	(50%–70% pred., *n* = 876)	1.23 (1.07–1.41)	0.0029	(*z*–score −2.5 to −4, *n* = 600)	1.3 (1.09–1.53)	0.0026
**FVC “severe”**	(<50% pred., *n* = 173)	1.76 (1.36–2.29)	<0.0001	(*z*–score < −4, *n* = 138)	1.58 (1.14–2.17)	0.0053
**TLCO (*z*-score)**		0.68 (0.66–0.7)	<0.0001		0.68 (0.65–0.7)	<0.0001
**Airway obstruction**		1.08 (0.9–1.29)	0.4108		1.07 (0.9–1.28)	0.4282
**Male sex**		1.69 (1.52–1.88)	<0.0001		1.68 (1.51–1.87)	<0.0001
**Age (years)**		1.06 (1.06–1.07)	<0.0001		1.06 (1.06–1.07)	<0.0001
**BMI (kg/m**^**2**^)		1.02 (1.01–1.03)	0.0023		1.02 (1.01–1.03)	0.0025
**CTD**		3.33 (2.69–4.12)	<0.0001		3.29 (2.66–4.08)	<0.0001
**HP**		2.25 (1.78–2.85)	<0.0001		2.23 (1.76–2.83)	<0.0001
**i-NSIP**		1.99 (1.39–2.86)	0.0002		1.96 (1.37–2.82)	0.0003
**IPF**		3.82 (3.08–4.73)	<0.0001		3.77 (3.04–4.68)	<0.0001
**o-ILD**		2.19 (1.78–2.7)	<0.0001		2.17 (1.76–2.67)	<0.0001
**u-ILD**		2.79 (2.09–3.73)	<0.0001		2.77 (2.07–3.7)	<0.0001
**Concordance (Harrell’s C-index)**		0.872 (0.864–0.880)			0.872 (0.864–0.880)	
	**Model with TLCO severity using 2005 guidelines (% predicted thresholds)**			**Model with TLCO severity using 2022 guidelines (*z*-score thresholds)**		
**TLCO “mild”**	(>60% pred., *n* = 1,445)	1.6 (1.35–1.9)	<0.0001	(*z*–score > –2.5, *n* = 1,012)	1.49 (1.24–1.79)	<0.0001
**TLCO “moderate”**	(40%–60% pred., *n* = 1,130)	2.42 (2.03–2.88)	<0.0001	(*z*–score −2.5 to −4, *n* = 1,124)	1.96 (1.65–2.34)	<0.0001
**TLCO “severe”**	(<40 % pred., *n* = 529)	5.59 (4.57–6.84)	<0.0001	(*z*–score < −4, *n* = 968)	4.11 (3.42–4.94)	<0.0001
**FVC (*z*-score)**		0.83 (0.79–0.88)	<0.0001		0.82 (0.78–0.86)	<0.0001
**Airway obstruction**		1.09 (0.91–1.3)	0.3479		1.1 (0.92–1.31)	0.2891
**Male sex**		1.5 (1.35–1.67)	<0.0001		1.64 (1.48–1.83)	<0.0001
**Age (years)**		1.06 (1.05–1.06)	<0.0001		1.06 (1.06–1.07)	<0.0001
**BMI (kg/m**^**2**^)		1.01 (1–1.02)	0.0665		1.01 (1–1.02)	0.0481
**CTD**		3.77 (3.03–4.68)	<0.0001		3.78 (3.05–4.68)	<0.0001
**HP**		2.78 (2.2–3.52)	<0.0001		2.68 (2.12–3.39)	<0.0001
**i-NSIP**		2.39 (1.66–3.42)	<0.0001		2.45 (1.71–3.51)	<0.0001
**IPF**		4.68 (3.77–5.82)	<0.0001		4.6 (3.71–5.71)	<0.0001
**o-ILD**		2.56 (2.08–3.15)	<0.0001		2.46 (2–3.03)	<0.0001
**u-ILD**		3.44 (2.58–4.58)	<0.0001		3.55 (2.67–4.73)	<0.0001
**Concordance (Harrell’s C-index)**		0.870 (0.862–0.878)			0.868 (0.860–0.876)	

BMI – body mass index; CI – confidence interval; CTD – connective tissue diseases pulmonary related disorders; FVC – forced vital capacity; HP – hypersensitivity pneumonitis; HR – hazard ratio; i-NSIP – idiopathic non-specific interstitial pneumonia; IPF – idiopathic pulmonary fibrosis; o-ILD – other ILDs; SAR – sarcoidosis; TLC – total lung capacity from body plethysmography; TLCO – lung transfer factor for carbon monoxide; u-ILD – unclassifiable interstitial lung disease;

In different models we used ventilatory indices and gas transfer expressed in % pred., *z*-scores and severity stages. Among the ventilation indices analyzed, FVC appeared to have the greatest impact on survival in the models (detailed data in, S1–S4 Models) with lung function as continuous values. The risk of death increase was 10.3% per one unit change of FVC *z*-score, compared to 8.1% and 8.2% for FEV_1_ and TLC, respectively. However, the impact of FVC was significantly weaker than that of TLCO, which showed an increase of risk over 30% for a one unit change of the *z*-score in every model.

When lung function was expressed as a percentage of predicted values, the risk of death increased by 8% and 35% for every 10% decrease in FVC and TLCO, respectively. Due to these findings, only the results of FVC and TLCO were considered in further analyses, which aligns with commonly accepted clinical practice. For the FVC severity stages model, TLCO severity (expressed as *z*-score) was included as a confounder, and similarly, for the TLCO severity stages model, FVC severity expressed using *z*-score was included as a confounder.

As suggested by the Kaplan–Meier analyses, both models using TLCO to grade severity provided equivalent mortality prediction. However, the 2005 classification of mildly low FVC (between 70% predicted and the LLN) was not associated with a significant increase in mortality when compared to patients with ILD with a normal FVC (see Table 5). The older the patient and the male sex, the higher the risk of mortality. The presence of airway obstruction was not an independent mortality risk factor except borderline result in model with TLCO and FVC expressed as *z*-scores. In all models, diagnosis of ILD other than sarcoidosis strongly affected the prognosis.

**Fig 3:**
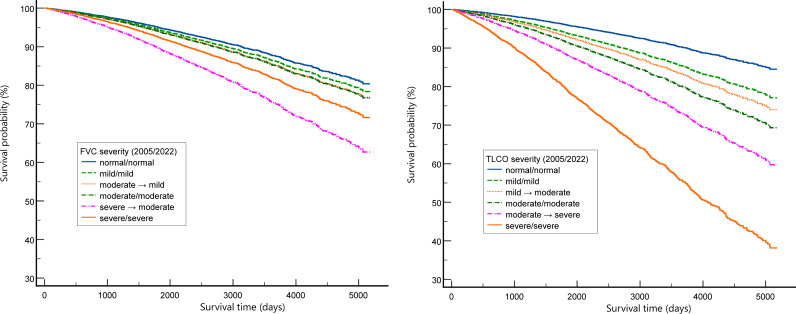
Cox proportional hazards model for severity of FVC (left panel) and TLCO (right panel) levels. Recategorized groups have the arrow in the middle of the name (ATS/ERS 2005 → ATS/ERS 2022). ATS – American Thoracic Society; ERS – European Respiratory Society; FVC – forced vital capacity; TLCO – lung transfer factor for carbon monoxide.

Using the same models, we compared consistent and non-consistent levels of severity grading with “normal” FVC and TLCO as reference. Results are presented in Fig 3, left and right panel respectively.

In both Cox regression models, age, male sex, all ILD diseases, and lung function (as continuous variables) were significant predictors of mortality (detailed data in S5 and S6 Models). In the model with FVC severity, survival was not significantly lower for patients classified as „mild” when compared to those with a normal FVC. There was a considerable overlap observed in the confidence intervals for the mild to moderate classes, with only the “severe → moderate” category significantly deviating from the rest and had HR bigger than “severe/severe”. In contrast, all TLCO severity classes were associated with increased risk of mortality. Both functional indices were significant predictors of mortality in Cox regression models when included as continuous variables (% pred. or *z*-score), however TLCO had much greater impact than FVC.

## Discussion

Our study is the large-scale analysis touching on the problem of reclassifying pulmonary dysfunction in patients with interstitial lung diseases in the context of their prognostic value. The comprehensive nature of the study (simultaneous spirometry, plethysmography and TLCO testing) performed in a single laboratory by experienced and trained in clinical trial staff is also an advantage.

The results of the pulmonary function tests obtained in different diagnostic groups are consistent with previous observations in the literature regarding the prevalence of obstruction in interstitial diseases [19,20]. It is noteworthy that restrictive disorders were not as prevalent as indicated by the reduced FVC value in most patients, except for those with IPF, i-NSIP, and u-ILD. The relatively reduced FVC compared to preserved TLC may be attributed to the phenotype of combined pulmonary fibrosis and emphysema (CPFE) [21].

PFTs are also used as eligibility criteria in many clinical trials, and, in some countries, as criteria for receiving financial support from national health systems for treatment.

The 2022 update of ATS/ERS PFT interpretation guidelines recommended the use of *z*-scores to define the LLNs, and also suggested *z*-score thresholds for mild, moderate, and severe categories for results below the LLN [4]. However, the 2022 severity thresholds were not disease specific and not based on morbidity or mortality. For example, a TLCO *z*-score below −4.0 is far below the predicted value, but this “severe” abnormality category was again not based on morbidity (such as dyspnea on exertion) and not based on an incremental risk of subsequent mortality for a group of patients with ILD. The relationship between % predicted values, the lower limit of normal expressed as % predicted, and *z*-scores has been extensively discussed in the literature, particularly in the context of the development of modern lung function reference equations [13–15] and their recommended application in clinical practice [4]. The aim of our study, however, was not to revisit these well-established theoretical concepts, but rather to assess the practical implications of transitioning from % predicted thresholds to *z*-score-based thresholds in risk stratification and mortality prediction in patients with interstitial lung diseases.

Our study demonstrates that the PFT interpretation of severity will change for a substantial number of patients with an ILD if the 2022 guidelines are followed instead of “sticking with” the 2005 guidelines. A previous study demonstrated this phenomenon for patients with airway obstruction [7]. In large populations of healthy adults, the average values of both FVC and TLCO decline linearly with age [14,15]. However, the absolute difference between the predicted value and the lower limit of normal (LLN), e.g., expressed in liters for FVC, remains relatively stable across age groups. This means that the range of values considered “normal” (i.e., the spread between the predicted value and the LLN) does not widen substantially with aging in absolute terms. When expressed as a percentage of predicted, however, the LLN declines from approximately 80% in younger adults (up to around 40 years of age) to approximately 70% at 80 years of age [15]. This increasing spread in % predicted values with age reflects the natural physiological decline in lung function over time.

Because *z*-scores adjust for this age-related change, they provide a more standardized method for expressing pulmonary function relative to age-appropriate norms. This is the primary reason ATS/ERS guidelines recommend the use of *z*-scores rather than % predicted when assessing pulmonary function, particularly in populations spanning a wide age range.

The recategorization of spirometric indices (FEV_1_ and FVC) resulted in a shift to a less severe category for the majority of recategorized patients, and this shift was associated with older age. The reason for this phenomenon is that the percent predicted lower limit of normal decreases with age which causes the same % predicted value to have a less negative *z*-score in older compared to younger subjects. The range of values considered normal (*z*-scores between −1.64 and +1.64) remains relatively constant across age groups when assessed in absolute values. Fig 1 effectively illustrates the phenomenon, demonstrating a considerable spread of results presented as *z*-scores for the same percentage value. For instance, an FVC of 50% predicted may correspond to a *z*-score of approximately −2.75 for an 80-year-old individual but as low as −4.2 for a 30-year-old individual. Conversely, a *z*-score −4 corresponds to approximately 50% of predicted in younger patients, but can be as low as 35% of predicted in older patients. Assessing severity based on *z*-score thresholds appear to align better with the clinical perspective. A 30-year-old with a 50% FVC value raises greater concerns compared to an 80-year-old with the same FVC percentage.

The relationship between % predicted and *z*-scores for TLCO follows a somewhat different pattern. The result cloud clearly has an arched shape (unlike in the case of FVC, where the dispersion is sheaf-shaped), although the distribution of cases by age is similar (younger individuals are shifted up/left). For this reason, the recategorization was for the younger ones, but in this case, sex also mattered (women were more frequently recategorized).

Our study confirms statements in current ILD guidelines: PFTs are an important element in the clinical assessment of these patients [18,22–24]. Both FVC and TLCO independently predict mortality in patients with IPF [1,2], however our study revealed that TLCO seems to be stronger predictor than FVC (35% versus 8% change in HR for every 10% difference in index and 30% versus 10% change for HR for every 1 *z*-score difference, respectively). We are aware that we did not include all other predictors of survival identified, for example, in IPF studies, such as 6-min walk distance, and desaturation during the 6-min walk test [25–27], but our intention was to show the phenomenon occurring in the entire group of diseases characterized by the potential of developing similar type of functional disorders (lung volume restriction and gas transfer disorders). It is also well known, that a change in functional parameters over time is a better predictor of survival than a single score in ILDs [28–31], but the main goal of this study is to test the hypothesis about how severity at first presentation is assessed and its relation to prognosis.

Kaplan–Meier survival analysis demonstrated a significant association between abnormally low lung function and all-cause mortality. Notably, our patients with ILD exhibited a distinct grouping of results consistent with the 2005 ATS/ERS severity categories for TLCO. However, caution must be exercised when drawing simplistic conclusions from these findings, as they may be misleading and lack full justification.

For instance, it may be tempting to conclude that patients with TLCO > 60% of the predicted value share a similar prognosis, regardless of whether their TLCO value expressed as a *z*-score falls above or below −2.5. Likewise, moderately low TLCO values (40% to 60% predicted) with a *z*-score below −4.0 (presently classified as “severe”), did not show a significant increase in mortality.

However, the aforementioned observations underwent notable changes when employing a multivariable Cox proportional hazards model. This model incorporated various factors, including age, sex, type of disease, and FVC expressed as a *z*-score. Utilizing univariate Kaplan–Meier survival analysis to compare severity scoring systems, particularly when one system retains age, sex, and size biases, can lead to erroneous conclusions.

S2 and S4 Models effectively illustrate this issue. When using percent of predicted values, the hazard ratio for lung function abnormality indicates minimal survival advantage for better function, and the hazards associated with sex and age are lower and significantly different from those observed using *z*-scores. Conversely, when assessing severity of function using *z*-scores, a stronger relationship with survival becomes apparent, with higher and more well-defined hazards for sex and age. This discrepancy arises because sex and age are encompassed within the percent predicted method, thus resulting in inherent biases. The authors are aware of other potential factors influencing the study results (e.g., comorbidities) that were not included in the models. It is also important to consider possible interactions between sex, age and diagnosis (IPF is known to affect older men more often, whereas sarcoidosis is a disease of a relatively younger population).

It is worth to note, that about 3/4th of patients had a normal FVC and about half had a normal TLCO, but most of them were diagnosed with sarcoidosis. Before the onset of their ILD, half of the patients had lung function above their predicted value. Change in lung function for an individual patient would be a much better predictor of subsequent morbidity and mortality [32], but very few patients have “baseline” PFTs measured as healthy young adults.

The presence of airway obstruction may be associated with a poor prognosis in some interstitial lung diseases [20] and therefore we have included the FEV_1_/FVC ratio in the analyses. In patients with asthma or COPD, the presence of airflow limitation (a low FEV_1_/FVC) and the percent predicted FEV_1_ are important when estimating disease severity, but although 13.2% of our patients with an ILD also had airflow limitation, it was not an independent predictor of all-cause mortality. Male sex and age were independent predictors of mortality, consistent with previous observations in lung fibrotic diseases [1,2].

Our study analyzed previously collected data from a single center, and thus was retrospective. Some patients received treatment, in accordance with the indications and therapeutic recommendations applicable at the time. However, for the majority of IPF patients, antifibrotic treatment only became available in Poland in 2017, meaning that most patients with IPF included in our analysis were not treated with antifibrotic drugs.

During the study period, diagnostic standards for interstitial lung diseases, including IPF, did not undergo any major revisions that would have significantly affected diagnostic criteria or disease classification. Therefore, we believe that changes in diagnostic practice are unlikely to have introduced substantial time-dependent bias in our cohort.

Furthermore, due to the relatively low prevalence of interstitial lung diseases in the general population, there is a lack of high-quality evidence demonstrating a significant impact of therapeutic interventions (including antifibrotic therapy) on overall mortality in this patient population. As a result, although supportive care was available, there is no clear evidence that it systematically improved survival outcomes, particularly among patients who may have been misclassified based on absolute pulmonary function thresholds.

Additionally, our results may not be generalizable to younger patients or those from non-Caucasian populations with ILD. Our primary outcome was all-cause mortality, as data on specific causes of death (e.g., accidental death, suicide, or deaths due to comorbidities) were not available. Finally, the statistical models used in this study do not account for all potential confounding factors, which may introduce some degree of uncertainty into our estimates.

The implementation of newly recommended severity stages for lung function impairment utilizing *z*-scores results in the reclassification of a substantial number of patients with interstitial lung diseases. Age plays a major role in this reclassification, with older patients being shifted to less severe categories for spirometric indices, while younger patients tend to be classified into more severe categories for TLCO. The adoption of reclassification and the use of *z*-scores appear to be justified due to their stronger association with overall prognosis. Notably, severe impairment of TLCO emerges as a significantly stronger predictor of mortality in ILDs compared to FVC within the same category. This highlights the importance of considering TLCO as a valuable marker in assessing disease severity and prognosis. Given the above considerations, the reasonable message, in our opinion, is that perhaps future clinical trials should be conducted using *z*-score-based classification rather than % predicted thresholds.

## Supporting information

S1 Table Numbers of patients presenting ventilatory and gas transfer disturbances. (PDF)

S2 Table Mean and median survival in groups according to diagnosis. (PDF)

S3 Table Numbers and percentages of deaths in each diagnosis category. (PDF)

S4 Table The hazard ratio (HR) and 95% confidence interval (95% CI) for mortality (sarcoidosis group as reference). (PDF)

S1 Fig Kaplan–Meier analyses for survival according to diagnosis. (CTD – connective tissue diseases pulmonary related disorders, HP – hypersensitivity pneumonitis, i-NSIP – idiopathic non-specific interstitial pneumonia, IPF – idiopathic pulmonary fibrosis, o-ILD – other ILDs, SAR – sarcoidosis, u-ILD – unclassifiable interstitial lung disease). Fourteen years of follow-up = 5,200 days.
(PDF)

S1 Model Sex, age, body mass index (BMI), the diagnosis group (sarcoidosis as the reference) and lung function: presence of airway obstruction, FEV_1_ (*z*-score), TLCO (*z*-score). (PDF)

S2 Model Sex, age, body mass index (BMI), the diagnosis group (sarcoidosis as the reference) and lung function: presence of airway obstruction, FVC (*z*-score), TLCO (*z*-score). (PDF)

S3 Model Sex, age, body mass index (BMI), the diagnosis group (sarcoidosis as the reference) and lung function: presence of airway obstruction, TLC (*z*-score), TLCO (*z*-score). (PDF)

S4 Model Sex, age, body mass index (BMI), the diagnosis group (sarcoidosis as the reference) and lung function: presence of airway obstruction, FVC (% predicted), TLCO (% predicted). (PDF)

S5 Model Sex, age, body mass index (BMI), the diagnosis group (sarcoidosis as the reference) and lung function: presence of airway obstruction, FVC severity (concordant and discordant, normal/normal as reference), TLCO (*z*-score). (PDF)

S6 Model Sex, age, body mass index (BMI), the diagnosis group (sarcoidosis as the reference) and lung function: presence of airway obstruction, TLCO severity (concordant and discordant, normal/normal as reference), FVC (*z*-score). (PDF)

S1 File STROBE checklist. (DOCX)

## Abbreviations

**:**
COPD: chronic obstructive pulmonary disease
CPFE: combined pulmonary fibrosis and emphysema
CTD: connective tissue disorders
FEV1: forced expiratory volume in one second
FVC: forced vital capacity
ILD: interstitial lung disease
i-NSIP: idiopathic non-specific interstitial pneumonia
IPF: idiopathic pulmonary fibrosis
LLN: lower limit of normal
PFTs: pulmonary function tests
SAR: sarcoidosis
TLC: total lung capacity
TLCO: transfer factor for carbon monoxide
u-ILD: unclassifiable interstitial lung disease
