# Supplementary material for: Assessment of lung function and severity grading in interstitial lung diseases (% predicted versus z-scores) and association with survival: A retrospective cohort study of 6,808 patients
Source: PLoS Med. 2025 May 29;22(5):e1004619. doi: 10.1371/journal.pmed.1004619 (PMC12121907; doi:10.1371/journal.pmed.1004619)
Supplement: S1 Table — (PDF) [file pmed.1004619.s001.pdf]

Supporting Information for:

Piotr W. Boros, Magdalena M. Martusewicz-Boros, Katarzyna B. Lewandowska.

Assessment of Lung Function and Severity Grading in Interstitial Lung Diseases (%Predicted vs Z-Scores) and Association with Survival: A Retrospective Cohort Study of 6,808 Patients.

S1 Table. Numbers of patients presenting ventilatory and gas transfer disturbances.

low TLCO	ventilatory_disturbance					
	**none**	**non-specific**	**pure obstructive**	**pure restrictive**	**mixed**	
**no**	3089<br>83.4% RT<br>69.1% CT<br>45.4% GT	80<br>2.2% RT<br>30.9% CT<br>1.2% GT	434<br>11.7% RT<br>52.9% CT<br>6.4% GT	90<br>2.4% RT<br>7.6% CT<br>1.3% GT	11<br>0.3% RT<br>14.1% CT<br>0.2% GT	3704 (54.4%)
**yes**	1379<br>44.4% RT<br>30.9% CT<br>20.3% GT	179<br>5.8% RT<br>69.1% CT<br>2.6% GT	387<br>12.5% RT<br>47.1% CT<br>5.7% GT	1092<br>35.2% RT<br>92.4% CT<br>16.0% GT	67<br>2.2% RT<br>85.9% CT<br>1.0% GT	3104 (45.6%)
	4468<br>65.60%	259<br>3.80%	821<br>12.10%	1182<br>17.40%	78<br>1.10%	6808
**Chi-squared**						1543.714
**DF**						4
**Significance level**						P < 0.0001

RT – % of row total, CT – % of column total, GT - % of grand total, non-specific: FEV1<LLN and TLC>LLN, FEV1/FVC>LLN, pure obstructive: FEV1/FVC<LLN and TLC>LLN, pure restrictive: TLC<LLN and FEV1/FVC>LLN, mixed: TLC<LLN and FEV1/FVC<LLN. DF – degrees of freedom; FEV1 – forced expiratory volume in 1 second; FVC – forced vital capacity; TLC – total lung capacity from body plethysmography; TLCO – lung transfer factor for carbon monoxide.
