# Supplementary material for: Assessment of lung function and severity grading in interstitial lung diseases (% predicted versus z-scores) and association with survival: A retrospective cohort study of 6,808 patients
Source: PLoS Med. 2025 May 29;22(5):e1004619. doi: 10.1371/journal.pmed.1004619 (PMC12121907; doi:10.1371/journal.pmed.1004619)
Supplement: S2 Table — (PDF) [file pmed.1004619.s002.pdf]

Piotr W. Boros, Magdalena M. Martusewicz-Boros, Katarzyna B. Lewandowska.

Assessment of Lung Function and Severity Grading in Interstitial Lung Diseases (%Predicted vs Z-Scores) and Association with Survival: A Retrospective Cohort Study of 6,808 Patients.

S2 Table. Mean and median survival in groups according to diagnosis.

Factor	Mean	SE	95% CI for the mean	Median	95% CI for the median
SAR	4993.266	14.816	4964.226 to 5022.306	-	
CTD	3303.705	78.992	3148.880 to 3458.530	3618.000	3210.000 to 3972.000
HP	3811.070	86.171	3642.176 to 3979.965	-	
i-NSIP	3575.122	190.438	3201.863 to 3948.381	-	
IPF	2091.157	59.672	1974.199 to 2208.114	1768.000	1612.000 to 1954.000
o-ILD	4220.823	51.481	4119.920 to 4321.725	-	
u-ILD	3184.044	145.727	2898.418 to 3469.669	4087.000	2487.000 to 4696.000
Overall	4202.050	21.917	4159.093 to 4245.007	-	

CI – confidence interval, CTD - connective tissue diseases pulmonary related disorders, HP - hypersensitivity pneumonitis, i-NSIP - idiopathic non-specific interstitial pneumonia, IPF - idiopathic pulmonary fibrosis, o-ILD - others ILDs, SAR – sarcoidosis, SE – standard error, u-ILD - unclassifiable interstitial lung disease
