# Supplementary material for: Assessment of lung function and severity grading in interstitial lung diseases (% predicted versus z-scores) and association with survival: A retrospective cohort study of 6,808 patients
Source: PLoS Med. 2025 May 29;22(5):e1004619. doi: 10.1371/journal.pmed.1004619 (PMC12121907; doi:10.1371/journal.pmed.1004619)
Supplement: S3 Table — (PDF) [file pmed.1004619.s003.pdf]

Piotr W. Boros, Magdalena M. Martusewicz-Boros, Katarzyna B. Lewandowska.

Assessment of Lung Function and Severity Grading in Interstitial Lung Diseases (%Predicted vs Z-Scores) and Association with Survival: A Retrospective Cohort Study of 6,808 Patients.

S3 Table. Numbers and percentages of deaths in each diagnosis category

Factor	Number of events [a]		Number censored [b]		Total sample size
	N	%	N	%	
SAR	160	5.03	3020	94.97	3180
CTD	288	45.00	352	55.00	640
HP	162	31.46	353	68.54	515
i-NSIP	40	31.25	88	68.75	128
IPF	532	70.28	225	29.72	757
o-ILD	263	18.91	1128	81.09	1391
u-ILD	80	40.61	117	59.39	197
Overall	1525	22.40	5283	77.60	6808

[a] survival = dead, [b] survival = alive, CTD - connective tissue diseases pulmonary related disorders, HP - hypersensitivity pneumonitis, i-NSIP - idiopathic non-specific interstitial pneumonia, IPF - idiopathic pulmonary fibrosis, o-ILD - others ILDs, SAR – sarcoidosis, u-ILD - unclassifiable interstitial lung disease
