# Supplementary material for: Assessment of lung function and severity grading in interstitial lung diseases (% predicted versus z-scores) and association with survival: A retrospective cohort study of 6,808 patients
Source: PLoS Med. 2025 May 29;22(5):e1004619. doi: 10.1371/journal.pmed.1004619 (PMC12121907; doi:10.1371/journal.pmed.1004619)
Supplement: S4 Table — (PDF) [file pmed.1004619.s004.pdf]

Piotr W. Boros, Magdalena M. Martusewicz-Boros, Katarzyna B. Lewandowska.

Assessment of Lung Function and Severity Grading in Interstitial Lung Diseases (%Predicted vs Z-Scores) and Association with Survival: A Retrospective Cohort Study of 6,808 Patients.

S4 Table. The hazard ratio (HR) and 95% confidence interval (95%CI) for mortality (sarcoidosis group as reference).

diagnosis	HR	95%CI
**CTD**	12.23	10.14 to 14.74
**HP**	8.04	6.58 to 9.82
**i-NSIP**	26.42	21.62 to 32.28
**IPF**	9.27	6.13 to 14.02
**o-ILD**	5.23	4.56 to 5.99
**SAR**	reference	
**u-ILD**	12.49	8.88 to 17.57

CI – confidence interval, CTD - connective tissue diseases pulmonary related disorders, HP - hypersensitivity pneumonitis, i-NSIP - idiopathic non-specific interstitial pneumonia, IPF - idiopathic pulmonary fibrosis, o-ILD - others ILDs, SAR – sarcoidosis, u-ILD - unclassifiable interstitial lung disease
