# Supplementary material for: Assessment of lung function and severity grading in interstitial lung diseases (% predicted versus z-scores) and association with survival: A retrospective cohort study of 6,808 patients
Source: PLoS Med. 2025 May 29;22(5):e1004619. doi: 10.1371/journal.pmed.1004619 (PMC12121907; doi:10.1371/journal.pmed.1004619)
Supplement: S1 Fig — (CTD – connective tissue diseases pulmonary related disorders, HP – hypersensitivity pneumonitis, i-NSIP – idiopathic non-specific interstitial pneumonia, IPF – idiopathic pulmonary fibrosis, o-ILD – other ILDs, SAR – sarcoidosis, u-ILD – unclassifiable interstitial lung disease). Fourteen years of follow-up = 5,200 days. (PDF) [file pmed.1004619.s005.pdf]

Supporting Information for:

Piotr W. Boros, Magdalena M. Martusewicz-Boros, Katarzyna B. Lewandowska.

Assessment of Lung Function and Severity Grading in Interstitial Lung Diseases (%Predicted vs Z-Scores) and Association with Survival: A Retrospective Cohort Study of 6,808 Patients.

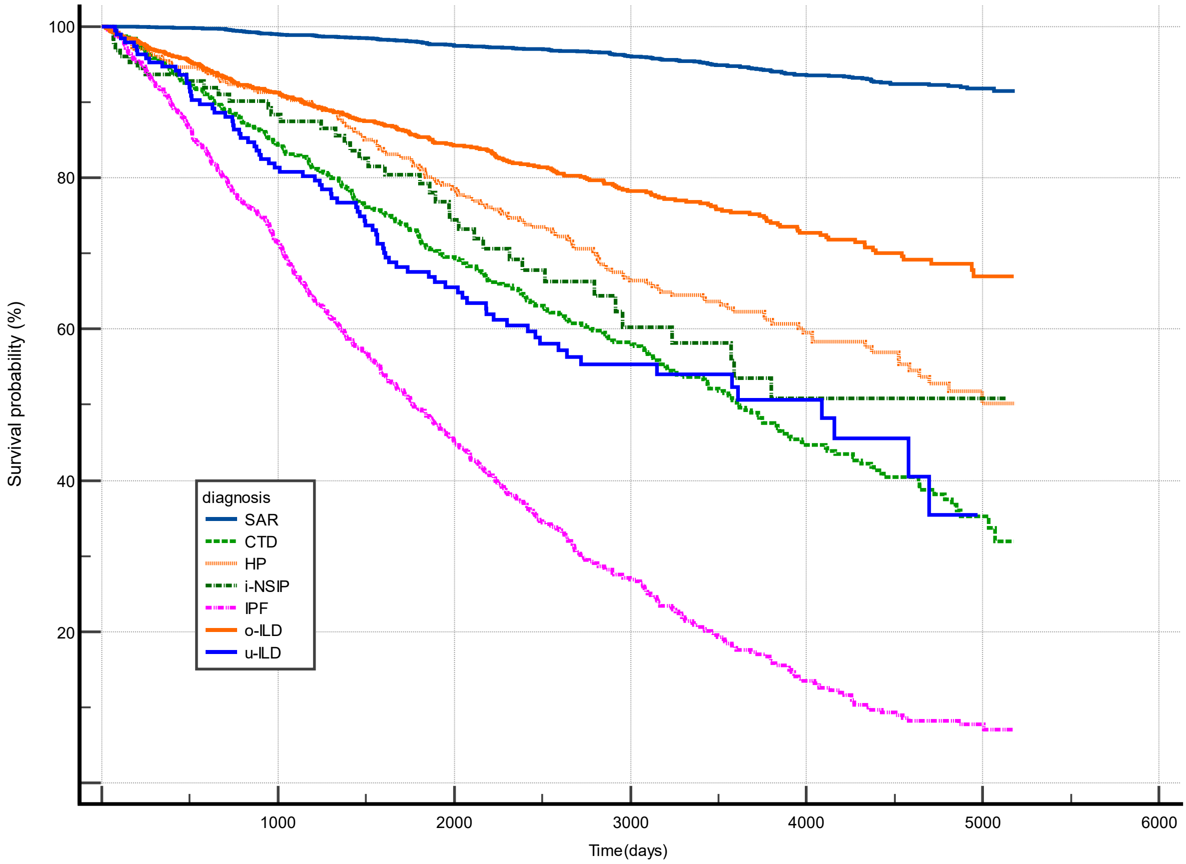

S1 Fig. Kaplan–Meier analyses for survival according to diagnosis (CTD - connective tissue diseases pulmonary related disorders, HP - hypersensitivity pneumonitis, i-NSIP - idiopathic non-specific interstitial pneumonia, IPF - idiopathic pulmonary fibrosis, o-ILD - others ILDs, SAR – sarcoidosis, u-ILD - unclassifiable interstitial lung disease). 14 years of follow-up = 5200 days.
