# Supplementary material for: Assessment of lung function and severity grading in interstitial lung diseases (% predicted versus z-scores) and association with survival: A retrospective cohort study of 6,808 patients
Source: PLoS Med. 2025 May 29;22(5):e1004619. doi: 10.1371/journal.pmed.1004619 (PMC12121907; doi:10.1371/journal.pmed.1004619)
Supplement: S1 Model — (PDF) [file pmed.1004619.s006.pdf]

Supporting Information for:

Piotr W. Boros, Magdalena M. Martusewicz-Boros, Katarzyna B. Lewandowska.

Assessment of Lung Function and Severity Grading in Interstitial Lung Diseases (%Predicted vs Z-Scores) and Association with Survival: A Retrospective Cohort Study of 6,808 Patients.

S1 Model. The Cox proportional hazards regression model: sex, age, body mass index (BMI), the diagnosis group (sarcoidosis as the reference) and lung function : presence of airway obstruction, FEV1 (z-score), TLCO (z-score)

Overall Model Fit

Null model -2 Log Likelihood	25489.084
Full model -2 Log Likelihood	22259.715
Chi-squared	3229.369
DF	12
Significance level	P < 0.0001

Concordance

Harrell's C-index	0.871
95% Confidence interval	0.863 to 0.879

Coefficients and Standard Errors

Covariate	b	SE	Wald	P	Exp(b)	95% CI of Exp(b)
age	0.05971	0.002581	535.3149	<0.0001	1.0615	1.0562 to 1.0669
sex="M"	0.5250	0.05433	93.3940	<0.0001	1.6905	1.5197 to 1.8804
bmi	0.01744	0.005668	9.4677	0.0021	1.0176	1.0064 to 1.0290
diagnosis_group="CTD"	1.2079	0.1089	122.9704	<0.0001	3.3463	2.7030 to 4.1427
diagnosis_group="HP"	0.8249	0.1200	47.2536	<0.0001	2.2817	1.8035 to 2.8868
diagnosis_group="i-NSIP"	0.6991	0.1845	14.3499	0.0002	2.0119	1.4013 to 2.8886
diagnosis_group="IPF"	1.3493	0.1096	151.5956	<0.0001	3.8549	3.1097 to 4.7785
diagnosis_group="o-ILD"	0.7718	0.1056	53.4491	<0.0001	2.1637	1.7593 to 2.6611
diagnosis_group="u-ILD"	1.0499	0.1480	50.3543	<0.0001	2.8574	2.1381 to 3.8186
airway_obstruction="yes"	-0.05592	0.09817	0.3245	0.5689	0.9456	0.7801 to 1.1462
fev1_z	-0.08417	0.02828	8.8557	0.0029	0.9193	0.8697 to 0.9717
tlco_z	-0.3941	0.01822	468.0477	<0.0001	0.6743	0.6506 to 0.6988

CI – confidence interval, CTD - connective tissue diseases pulmonary related disorders, DF – degrees of freedom, HP - hypersensitivity pneumonitis, i-NSIP - idiopathic non-specific interstitial pneumonia, IPF - idiopathic pulmonary fibrosis, o-ILD - others ILDs, SAR – sarcoidosis, SE – standard error, u-ILD - unclassifiable interstitial lung disease, FEV1 – forced expiratory volume in 1 second, TLCO – lung transfer factor for carbon monoxide.
