# Supplementary material for: Assessment of lung function and severity grading in interstitial lung diseases (% predicted versus z-scores) and association with survival: A retrospective cohort study of 6,808 patients
Source: PLoS Med. 2025 May 29;22(5):e1004619. doi: 10.1371/journal.pmed.1004619 (PMC12121907; doi:10.1371/journal.pmed.1004619)
Supplement: S2 Model — (PDF) [file pmed.1004619.s007.pdf]

Piotr W. Boros, Magdalena M. Martusewicz-Boros, Katarzyna B. Lewandowska.

Assessment of Lung Function and Severity Grading in Interstitial Lung Diseases (%Predicted vs Z-Scores) and Association with Survival: A Retrospective Cohort Study of 6,808 Patients.

S2 Model. The Cox proportional hazards regression model: sex, age, body mass index (BMI), the diagnosis group (sarcoidosis as the reference) and lung function : presence of airway obstruction, FVC (z-score), TLCO (z-score)

Overall Model Fit

Null model -2 Log Likelihood	25489.084
Full model -2 Log Likelihood	22251.423
Chi-squared	3237.662
DF	12
Significance level	P < 0.0001

Concordance

Harrell's C-index	0.872
95% Confidence interval	0.864 to 0.880

Coefficients and Standard Errors

Covariate	b	SE	Wald	P	Exp(b)	95% CI of Exp(b)
age	0.06041	0.002557	558.3448	<0.0001	1.0623	1.0570 to 1.0676
sex="M"	0.5212	0.05432	92.0917	<0.0001	1.6841	1.5140 to 1.8733
bmi	0.01661	0.005663	8.6044	0.0034	1.0168	1.0055 to 1.0281
diagnosis_group="CTD"	1.1941	0.1090	120.0550	<0.0001	3.3007	2.6659 to 4.0868
diagnosis_group="HP"	0.8085	0.1201	45.3377	<0.0001	2.2446	1.7739 to 2.8403
diagnosis_group="i-NSIP"	0.6814	0.1846	13.6296	0.0002	1.9766	1.3766 to 2.8381
diagnosis_group="IPF"	1.3353	0.1094	148.9197	<0.0001	3.8011	3.0674 to 4.7104
diagnosis_group="o-ILD"	0.7757	0.1055	54.0334	<0.0001	2.1722	1.7663 to 2.6713
diagnosis_group="u-ILD"	1.0296	0.1479	48.4513	<0.0001	2.7999	2.0953 to 3.7415
airway_obstruction="yes"	0.07932	0.09040	0.7700	0.3802	1.0826	0.9068 to 1.2924
fvc_z	-0.1090	0.02639	17.0444	<0.0001	0.8968	0.8516 to 0.9444
tlco_z	-0.3795	0.01866	413.5100	<0.0001	0.6842	0.6596 to 0.7097

CI – confidence interval, CTD - connective tissue diseases pulmonary related disorders, DF – degrees of freedom, HP - hypersensitivity pneumonitis, i-NSIP - idiopathic non-specific interstitial pneumonia, IPF - idiopathic pulmonary fibrosis, o-ILD - others ILDs, SAR – sarcoidosis, SE – standard error, u-ILD - unclassifiable interstitial lung disease, FVC – forced vital capacity, TLCO – lung transfer factor for carbon monoxide.
