# Supplementary material for: Assessment of lung function and severity grading in interstitial lung diseases (% predicted versus z-scores) and association with survival: A retrospective cohort study of 6,808 patients
Source: PLoS Med. 2025 May 29;22(5):e1004619. doi: 10.1371/journal.pmed.1004619 (PMC12121907; doi:10.1371/journal.pmed.1004619)
Supplement: S3 Model — (PDF) [file pmed.1004619.s008.pdf]

Supporting Information for:

Piotr W. Boros, Magdalena M. Martusewicz-Boros, Katarzyna B. Lewandowska.

Assessment of Lung Function and Severity Grading in Interstitial Lung Diseases (%Predicted vs Z-Scores) and Association with Survival: A Retrospective Cohort Study of 6,808 Patients.

S3 Model. The Cox proportional hazards regression model: sex, age, body mass index (BMI), the diagnosis group (sarcoidosis as the reference) and lung function : presence of airway obstruction, TLC (z-score), TLCO (z-score).

Overall Model Fit

Null model -2 Log Likelihood	25489.084
Full model -2 Log Likelihood	22255.709
Chi-squared	3233.375
DF	12
Significance level	P < 0.0001

Concordance

Harrell's C-index	0.872
95% Confidence interval	0.864 to 0.880

Coefficients and Standard Errors

Covariate	b	SE	Wald	P	Exp(b)	95% CI of Exp(b)
age	0.05823	0.002444	567.6524	<0.0001	1.0600	1.0549 to 1.0651
sex="M"	0.4906	0.05527	78.8025	<0.0001	1.6334	1.4657 to 1.8202
bmi	0.01570	0.005751	7.4555	0.0063	1.0158	1.0044 to 1.0273
diagnosis_group="CTD"	1.2169	0.1093	124.0010	<0.0001	3.3766	2.7256 to 4.1831
diagnosis_group="HP"	0.8482	0.1205	49.5689	<0.0001	2.3355	1.8443 to 2.9576
diagnosis_group="i-NSIP"	0.6921	0.1847	14.0465	0.0002	1.9980	1.3912 to 2.8694
diagnosis_group="IPF"	1.3422	0.1098	149.4904	<0.0001	3.8275	3.0865 to 4.7463
diagnosis_group="o-ILD"	0.8130	0.1063	58.5163	<0.0001	2.2546	1.8306 to 2.7767
diagnosis_group="u-ILD"	1.0399	0.1482	49.2421	<0.0001	2.8291	2.1159 to 3.7826
airway_obstruction="yes"	0.1924	0.09739	3.9030	0.0482	1.2122	1.0015 to 1.4671
tlc_z	-0.08532	0.02378	12.8713	0.0003	0.9182	0.8764 to 0.9620
tlco_z	-0.3867	0.01852	435.8597	<0.0001	0.6793	0.6551 to 0.7044

CI – confidence interval, CTD - connective tissue diseases pulmonary related disorders, DF – degrees of freedom, HP - hypersensitivity pneumonitis, i-NSIP - idiopathic non-specific interstitial pneumonia, IPF - idiopathic pulmonary fibrosis, o-ILD - others ILDs, SAR – sarcoidosis, SE – standard error, u-ILD - unclassifiable interstitial lung disease, TLC – total lung capacity, TLCO – lung transfer factor for carbon monoxide.
