# Supplementary material for: Assessment of lung function and severity grading in interstitial lung diseases (% predicted versus z-scores) and association with survival: A retrospective cohort study of 6,808 patients
Source: PLoS Med. 2025 May 29;22(5):e1004619. doi: 10.1371/journal.pmed.1004619 (PMC12121907; doi:10.1371/journal.pmed.1004619)
Supplement: S4 Model — (PDF) [file pmed.1004619.s009.pdf]

Piotr W. Boros, Magdalena M. Martusewicz-Boros, Katarzyna B. Lewandowska.

Assessment of Lung Function and Severity Grading in Interstitial Lung Diseases (%Predicted vs Z-Scores) and Association with Survival: A Retrospective Cohort Study of 6,808 Patients.

S4 Model. The Cox proportional hazards regression model: sex, age, body mass index (BMI), the diagnosis group (sarcoidosis as the reference) and lung function : presence of airway obstruction, FVC (%predicted), TLCO (%predicted).

Overall Model Fit

Null model -2 Log Likelihood	25489.084
Full model -2 Log Likelihood	22292.952
Chi-squared	3196.132
DF	12
Significance level	P < 0.0001

Concordance

Harrell's C-index	0.870
95% Confidence interval	0.862 to 0.878

Coefficients and Standard Errors

Covariate	b	SE	Wald	P	Exp(b)	95% CI of Exp(b)
age	0.04945	0.002473	399.9003	<0.0001	1.0507	1.0456 to 1.0558
sex="M"	0.3727	0.05413	47.3913	<0.0001	1.4516	1.3055 to 1.6141
bmi	0.01588	0.005667	7.8562	0.0051	1.0160	1.0048 to 1.0274
diagnosis_group="CTD"	1.1933	0.1094	118.9929	<0.0001	3.2979	2.6615 to 4.0865
diagnosis_group="HP"	0.8256	0.1201	47.2871	<0.0001	2.2833	1.8045 to 2.8891
diagnosis_group="i-NSIP"	0.7139	0.1839	15.0750	0.0001	2.0420	1.4241 to 2.9280
diagnosis_group="IPF"	1.3366	0.1101	147.2753	<0.0001	3.8062	3.0672 to 4.7233
diagnosis_group="o-ILD"	0.7781	0.1058	54.0808	<0.0001	2.1773	1.7695 to 2.6791
diagnosis_group="u-ILD"	1.0645	0.1478	51.8966	<0.0001	2.8993	2.1703 to 3.8732
airway_obstruction="yes"	0.06260	0.09050	0.4785	0.4891	1.0646	0.8916 to 1.2712
fvc_pp	-0.007874	0.001801	19.1212	<0.0001	0.9922	0.9887 to 0.9957
tlco_pp	-0.03515	0.001848	361.8914	<0.0001	0.9655	0.9620 to 0.9690

CI – confidence interval, CTD - connective tissue diseases pulmonary related disorders, DF – degrees of freedom, HP - hypersensitivity pneumonitis, i-NSIP - idiopathic non-specific interstitial pneumonia, IPF - idiopathic pulmonary fibrosis, o-ILD - others ILDs, SAR – sarcoidosis, SE – standard error, u-ILD - unclassifiable interstitial lung disease, FVC – forced vital capacity, TLCO – lung transfer factor for carbon monoxide.
