# Supplementary material for: Assessment of lung function and severity grading in interstitial lung diseases (% predicted versus z-scores) and association with survival: A retrospective cohort study of 6,808 patients
Source: PLoS Med. 2025 May 29;22(5):e1004619. doi: 10.1371/journal.pmed.1004619 (PMC12121907; doi:10.1371/journal.pmed.1004619)
Supplement: S5 Model — (PDF) [file pmed.1004619.s010.pdf]

Piotr W. Boros, Magdalena M. Martusewicz-Boros, Katarzyna B. Lewandowska.

Assessment of Lung Function and Severity Grading in Interstitial Lung Diseases (%Predicted vs Z-Scores) and Association with Survival: A Retrospective Cohort Study of 6,808 Patients.

S5 Model. The Cox proportional hazards regression model: sex, age, body mass index (BMI), the diagnosis group (sarcoidosis as the reference) and lung function : presence of airway obstruction, FVC severity (concordant and discordant, normal/normal as reference), TLCO (z-score).

Overall Model Fit

Null model -2 Log Likelihood	25413.075
Full model -2 Log Likelihood	22168.941
Chi-squared	3244.134
DF	16
Significance level	P < 0.0001

Concordance

Harrell's C-index	0.872
95% Confidence interval	0.864 to 0.880

Coefficients and Standard Errors

Covariate	b	SE	Wald	P	Exp(b)	95% CI of Exp(b)
FVC "2_mild/2_mild"	0.1123	0.1033	1.1827	0.2768	1.1189	0.9138 to 1.3699
FVC "3_moderate/2_mild"	0.1900	0.08598	4.8820	0.0271	1.2092	1.0217 to 1.4312
FVC "3_moderate/3_moderate"	0.1973	0.08963	4.8468	0.0277	1.2181	1.0219 to 1.4521
FVC "4_severe/3_moderate"	0.7629	0.1776	18.4420	<0.0001	2.1445	1.5139 to 3.0376
FVC "4_severe/4_severe"	0.4259	0.1675	6.4674	0.0110	1.5310	1.1026 to 2.1258
tlco_z	-0.3878	0.01818	454.7478	<0.0001	0.6786	0.6548 to 0.7032
airway_obstruction="yes"	0.06584	0.09090	0.5248	0.4688	1.0681	0.8938 to 1.2763
sex="M"	0.5289	0.05454	94.0617	<0.0001	1.6971	1.5251 to 1.8886
age	0.05956	0.002685	491.8858	<0.0001	1.0614	1.0558 to 1.0670
bmi	0.01698	0.005701	8.8667	0.0029	1.0171	1.0058 to 1.0286
diagnosis_group="CTD"	1.2193	0.1097	123.5197	<0.0001	3.3848	2.7299 to 4.1969
diagnosis_group="HP"	0.8130	0.1211	45.0907	<0.0001	2.2547	1.7784 to 2.8585
diagnosis_group="i-NSIP"	0.7068	0.1852	14.5621	0.0001	2.0274	1.4102 to 2.9147
diagnosis_group="IPF"	1.3505	0.1101	150.4845	<0.0001	3.8593	3.1103 to 4.7887
diagnosis_group="o-ILD"	0.7926	0.1062	55.7412	<0.0001	2.2091	1.7941 to 2.7201
diagnosis_group="u-ILD"	1.0317	0.1484	48.3339	<0.0001	2.8059	2.0977 to 3.7532

CI – confidence interval, CTD - connective tissue diseases pulmonary related disorders, DF – degrees of freedom, HP - hypersensitivity pneumonitis, i-NSIP - idiopathic non-specific interstitial pneumonia, IPF - idiopathic pulmonary fibrosis, o-ILD - others ILDs, SAR – sarcoidosis, SE – standard error, u-ILD - unclassifiable interstitial lung disease, FVC – forced vital capacity, TLCO – lung transfer factor for carbon monoxide.
