# Supplementary material for: Assessment of lung function and severity grading in interstitial lung diseases (% predicted versus z-scores) and association with survival: A retrospective cohort study of 6,808 patients
Source: PLoS Med. 2025 May 29;22(5):e1004619. doi: 10.1371/journal.pmed.1004619 (PMC12121907; doi:10.1371/journal.pmed.1004619)
Supplement: S6 Model — (PDF) [file pmed.1004619.s011.pdf]

Piotr W. Boros, Magdalena M. Martusewicz-Boros, Katarzyna B. Lewandowska.

Assessment of Lung Function and Severity Grading in Interstitial Lung Diseases (%Predicted vs Z-Scores) and Association with Survival: A Retrospective Cohort Study of 6,808 Patients.

S6 Model. The Cox proportional hazards regression model: sex, age, body mass index (BMI), the diagnosis group (sarcoidosis as the reference) and lung function : presence of airway obstruction, TLCO severity (concordant and discordant, normal/normal as reference), FVC (z-score).

Overall Model Fit

Null model -2 Log Likelihood	25413.075
Full model -2 Log Likelihood	22250.374
Chi-squared	3162.701
DF	16
Significance level	P < 0.0001

Concordance

Harrell's C-index	0.871
95% Confidence interval	0.863 to 0.879

Coefficients and Standard Errors

Covariate	b	SE	Wald	P	Exp(b)	95% CI of Exp(b)
TLCO "2_mild/2_mild"	0.4294	0.09446	20.6630	<0.0001	1.5363	1.2767 to 1.8488
TLCO "2_mild/3_moderate"	0.5815	0.1214	22.9618	<0.0001	1.7887	1.4101 to 2.2690
TLCO "3_moderate/3_moderate"	0.7753	0.09530	66.1735	<0.0001	2.1712	1.8012 to 2.6171
TLCO "3_moderate/4_severe"	1.1216	0.1079	108.0119	<0.0001	3.0697	2.4844 to 3.7927
TLCO "4_severe/4_severe"	1.7452	0.1033	285.5522	<0.0001	5.7269	4.6774 to 7.0118
fvc_z	-0.1691	0.02596	42.4380	<0.0001	0.8444	0.8025 to 0.8885
airway_obstruction="yes"	0.08500	0.09084	0.8757	0.3494	1.0887	0.9112 to 1.3009
sex="M"	0.4422	0.05496	64.7455	<0.0001	1.5561	1.3972 to 1.7331
age	0.05648	0.002619	465.2760	<0.0001	1.0581	1.0527 to 1.0636
bmi	0.01119	0.005731	3.8123	0.0509	1.0113	1.0000 to 1.0227
diagnosis_group="CTD"	1.3238	0.1107	142.9511	<0.0001	3.7576	3.0246 to 4.6683
diagnosis_group="HP"	1.0000	0.1209	68.3650	<0.0001	2.7182	2.1446 to 3.4454
diagnosis_group="i-NSIP"	0.8705	0.1847	22.2076	<0.0001	2.3881	1.6627 to 3.4300
diagnosis_group="IPF"	1.5331	0.1110	190.8854	<0.0001	4.6325	3.7270 to 5.7580
diagnosis_group="o-ILD"	0.9264	0.1064	75.7609	<0.0001	2.5254	2.0499 to 3.1112
diagnosis_group="u-ILD"	1.2276	0.1472	69.5654	<0.0001	3.4132	2.5578 to 4.5546

CI – confidence interval, CTD - connective tissue diseases pulmonary related disorders, DF – degrees of freedom, HP - hypersensitivity pneumonitis, i-NSIP - idiopathic non-specific interstitial pneumonia, IPF - idiopathic pulmonary fibrosis, o-ILD - others ILDs, SAR – sarcoidosis, SE – standard error, u-ILD - unclassifiable interstitial lung disease, FVC – forced vital capacity, TLCO – lung transfer factor for carbon monoxide.
